# Longitudinal PET/CT-Derived Body Composition Changes During Systemic Therapy in Patients with Lung Cancer

**DOI:** 10.3390/diagnostics16182930

**Published:** 2026-09-10

**Authors:** Bojun Wang, Song Xue, Jueya Zhang, Yuqi Wang, Hanfei Zhang, Chengwen Zheng, Huiyuan Zhang, Yang Yang, Marcus Hacker, Xiang Li

**Affiliations:** 1 Beijing Tuberculosis and Thoracic Tumor Research Institute, Department of Nuclear Medicine, Beijing Chest Hospital, Capital Medical University, Beijing 101100, China; 2 Division of Nuclear Medicine, Department of Biomedical Imaging and Image-Guided Therapy, Vienna General Hospital, Medical University of Vienna, 1090 Vienna, Austria

**Keywords:** lung cancer, ^18^F-FDG PET/CT, body composition, torso fat, progression-free survival, metabolic network

## Abstract

**Purpose**: Host metabolic status may affect outcomes in lung cancer, but longitudinal changes in body composition and systemic metabolism remain incompletely understood. We examined whether paired ^18^F-FDG PET/CT-derived host features were associated with progression-free survival (PFS) during systemic therapy. **Methods**: Fifty-three patients with confirmed lung cancer who underwent baseline and post-treatment ^18^F-FDG PET/CT were retrospectively included. Tumor metabolic burden, body composition, organ metabolism, and brain metabolism were quantified. Delta values were defined as post-treatment minus baseline measurements. Associations with PFS were assessed using Kaplan–Meier analysis, adjusted Cox regression and exploratory metabolic network analysis. **Results**: During follow-up, 31 patients experienced disease progression, and median PFS was 7 months. SM volume (*p* = 0.001) decreased, and MedF volume (*p* < 0.001) increased at follow-up after FDR adjustment, whereas the numerical increases in SAT and TF volumes did not remain statistically significant after correction for multiple comparisons. Higher delta torso fat (TF) volume was associated with a lower risk of progression after adjustment (HR = 0.64, 95% CI: 0.43–0.97, *p* = 0.033). In the temporally aligned analysis of 29 patients whose follow-up PET/CT preceded progression, delta TF volume was not associated with subsequent PFS (HR, 1.84; 95% CI, 0.91–3.68; *p* = 0.087). Total network perturbation strength decreased after treatment (*p* = 0.002) but was not associated with PFS. **Conclusions**: Paired ^18^F-FDG PET/CT characterized longitudinal changes in body composition during systemic therapy. Delta TF volume showed an adjusted association with PFS in the exploratory full-cohort model, but its prognostic relevance requires further validation.

## 1. Introduction

Lung cancer remains a major contributor to the global cancer burden. According to Global Cancer Observatory 2022 estimates, lung cancer was the most commonly diagnosed malignancy in men worldwide and one of the leading causes of cancer-related death in both men and women [1]. Systemic treatment strategies, particularly immune checkpoint inhibitors and molecularly targeted therapies, have substantially changed the clinical management of lung cancer in recent years. Even so, treatment benefit varies widely between patients.

Conventional prognostic assessment mainly relies on tumor-related factors, including tumor-node-metastasis (TNM) stage, programmed death-ligand 1 (PD-L1) expression, and tumor metabolic burden. These factors have clinical value, but they do not fully explain differences in treatment response and survival. Host-related factors, including nutritional reserve, adiposity, skeletal muscle status, and systemic metabolic condition, may also influence the prognosis of patients with cancer. In patients with advanced non-small-cell lung cancer (NSCLC) treated with immune checkpoint inhibitors, higher body mass index (BMI) has been associated with improved survival in some studies, suggesting that metabolic status may modify treatment outcomes [2]. However, the relationship between obesity and cancer prognosis is complex. Excess adiposity may also reflect chronic inflammation, metabolic dysfunction, and impaired immune regulation [3]. These findings suggest that treatment benefit may depend on tumor burden as well as body composition and systemic metabolic status.

BMI is widely used as a clinical indicator of obesity, but it has important limitations as a marker of metabolic status. BMI cannot distinguish adipose tissue from skeletal muscle, describe fat distribution, or determine whether body weight changes reflect visceral adiposity, subcutaneous adiposity, or muscle wasting. These limitations are particularly relevant in patients with cancer, because body weight and BMI may remain relatively stable despite clinically meaningful changes in skeletal muscle and adipose tissue. Cancer cachexia and treatment-related wasting are characterized by progressive skeletal muscle loss, with or without loss of fat mass, and are closely associated with metabolic dysfunction and systemic inflammation [4,5]. According to the revised European Working Group on Sarcopenia in Older People 2 consensus, low muscle strength is the primary parameter of sarcopenia, whereas low muscle quantity or quality confirms the diagnosis [6]. Therefore, imaging-derived skeletal muscle measurements reflect muscle quantity but do not assess muscle strength.

Compared with BMI, imaging-derived body composition provides a more direct and region-specific assessment of the host phenotype. PET/CT-based body composition analysis allows quantitative assessment of skeletal muscle, subcutaneous adipose tissue, torso fat, and regional adipose depots at the individual level. Recent studies in advanced NSCLC have shown that imaging-derived body composition parameters are associated with immunotherapy outcomes, suggesting that body composition may provide prognostic information beyond BMI alone [7,8]. However, most previous imaging studies have relied on baseline body composition measurements from a single pretreatment scan. Tumor burden, nutritional status, systemic inflammation, and metabolic status may all change during lung cancer treatment. Longitudinal changes in body composition may therefore reflect host metabolic adaptation more accurately than a single baseline measurement.

^18^F-fluorodeoxyglucose PET/CT (^18^F-FDG PET/CT) is routinely used for staging, treatment response assessment, and prognostic evaluation in lung cancer. Beyond tumor imaging, a single PET/CT examination can provide tumor metabolic burden, organ glucose metabolism, and body composition. Tumor metabolic parameters have prognostic value in lung cancer, but they mainly reflect tumor-side disease burden [9]. In contrast, longitudinal changes in body composition may reflect host metabolic reserve and treatment-related systemic change. Paired pre-treatment and post-treatment PET/CT imaging can evaluate host metabolic remodeling during therapy.

Cancer is increasingly recognized as a systemic disease. Tumor progression and treatment affect distant organs, skeletal muscle, and adipose tissue and may alter immune regulation and whole-body energy metabolism [4]. In this context, a single imaging biomarker may not fully capture systemic responses to treatment. Network-based analysis offers an exploratory approach to characterizing relationships among organs, body composition compartments, and metabolism-related brain regions. In this study, we used a PET/CT-derived multiorgan metabolic network to investigate these relationships. Each imaging-derived measure from an organ, brain region, or body composition compartment was represented as a network node, and the strength of network perturbation was used to summarize individual deviations from the baseline reference network. This approach complements conventional body composition analysis by assessing whether changes in individual tissue compartments occur as part of broader treatment-related remodeling of coordinated metabolism across the body. We therefore used it as an exploratory method to characterize systemic metabolic remodeling and examine its association with PFS.

This study investigated the prognostic value of metabolism-related imaging biomarkers derived from paired pre-treatment and post-treatment ^18^F-FDG PET/CT in lung cancer patients receiving systemic treatment. In addition, we constructed a metabolic network to quantify treatment-related systemic metabolic remodeling and explored the association between network features and PFS.

## 2. Methods

### 2.1. Participants

We retrospectively screened 3237 patients who underwent ^18^F-FDG PET/CT in the Department of Nuclear Medicine of Beijing Chest Hospital between June 2023 and March 2026. Patients with pathologically confirmed lung cancer who underwent at least two ^18^F-FDG PET/CT examinations were screened.

**Figure 1:**
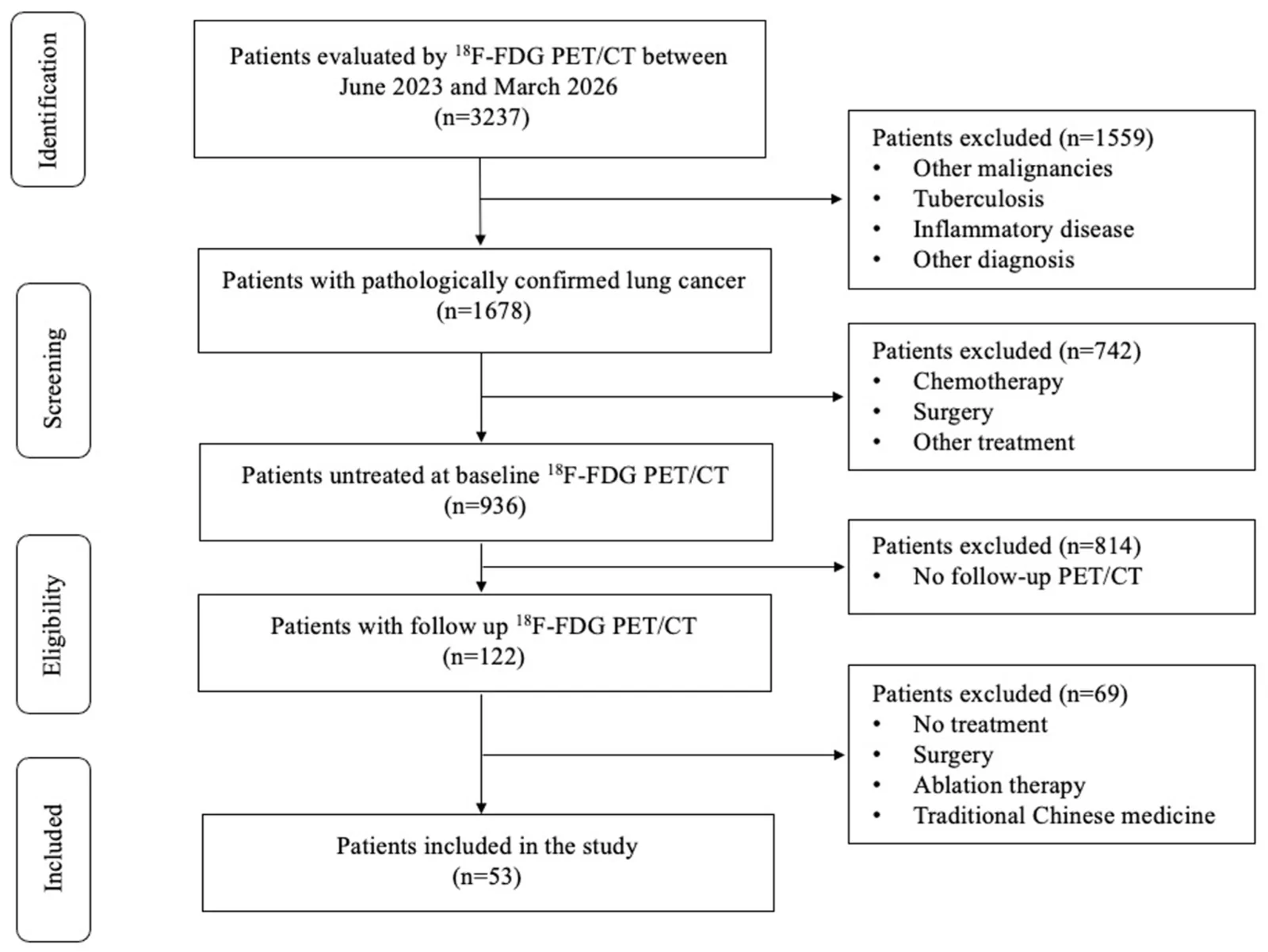
Flowchart of patient selection. Note: PET/CT: positron emission tomography/computed tomography.

The inclusion criteria were as follows: (1) pathologically confirmed primary lung adenocarcinoma (ADC), squamous cell carcinoma (SCC), or small-cell lung cancer (SCLC); (2) no anticancer treatment before baseline ^18^F-FDG PET/CT; (3) receipt of systemic therapy between baseline and follow-up PET/CT, including targeted therapy alone, chemotherapy plus immunotherapy, or targeted therapy plus immunotherapy; and (4) completion of both baseline and follow-up ^18^F-FDG PET/CT examinations. Patients were excluded if they had another malignancy, pulmonary tuberculosis, or an active inflammatory disease, or if they received no systemic therapy or underwent surgery, radiotherapy, ablation, or other local anticancer treatment between the two PET/CT examinations. The patient selection process and reasons for exclusion are summarized in Figure 1.

### 2.2. Clinical Data and Outcome Definition

Patients with lung cancer who received systemic treatment and completed baseline and follow-up ^18^F-FDG PET/CT examinations were included. Baseline PET/CT was defined as the examination performed before the initiation of systemic therapy. Follow-up PET/CT was defined as the first PET/CT examination performed after baseline. Clinical variables included age, sex, BMI, TNM stage and pathological type. TNM staging was assigned according to the 8th edition of the AJCC/UICC staging system. The data cutoff was April 2026.

Progression-free survival (PFS) was defined as the time from the date of the baseline PET/CT examination to disease progression or death. Patients without progression or death were censored at the last follow-up. The event indicator was coded as 1 for progression or death and 0 for censoring. Disease progression was determined retrospectively from serial CT examinations, supplemented by PET/CT findings, using routine RECIST-based radiological assessment.

### 2.3. PET/CT Acquisition

PET/CT imaging was performed in the Department of Nuclear Medicine, Beijing Chest Hospital, Capital Medical University, using a uMI 780 PET/CT scanner (United Imaging Healthcare, Shanghai, China). All participants fasted for 6 h before PET/CT examination. PET/CT was performed only when the fasting blood glucose level was ≤11.1 mmol/L. After intravenous administration of ^18^F-FDG at 3.7 MBq/kg body weight, participants rested for 60 min in a quiet, temperature-controlled environment and were instructed to drink water. After the uptake period, participants were positioned supine on the scanner bed and imaged in the head-first position. Whole-body PET/CT acquisition extended from the skull base to the mid-thigh.

CT was performed using a helical acquisition mode, with a tube voltage of 120 kVp, tube current of 130 mA, slice thickness of 3 mm, and interslice spacing of 1.5 mm. PET acquisition was performed with an energy window of 430 to 1000 keV and a single-frame acquisition duration of 180 s. PET images were reconstructed using three-dimensional iterative reconstruction incorporating time-of-flight (TOF) information and point-spread function (PSF) modeling. The reconstructed PET images had a matrix size of 150 × 150, an in-plane voxel size of 4.0 mm × 4.0 mm, and a slice thickness of 2.68 mm. CT-based attenuation correction, random coincidence correction, scatter correction, and radioactive decay correction were applied during image processing. Baseline and post-treatment PET/CT examinations were performed using the same acquisition and reconstruction protocol.

### 2.4. Image Segmentation and Feature Extraction

Raw PET/CT DICOM data were first converted to NIfTI format. PET images were converted to body weight-normalized standardized uptake value (SUV) images. The injected dose was corrected for radioactive decay before SUV calculation. All quantitative parameters were extracted from SUV-normalized PET images and reviewed segmentation masks.

^18^F-FDG PET/CT images were used to derive tumor SUV_max_, metabolic tumor volume (MTV) and total lesion glycolysis (TLG). Tumor lesions on whole-body ^18^F-FDG PET images were segmented using an artificial intelligence-assisted workflow based on nnU-Net. An experienced nuclear medicine physician visually reviewed and corrected the initial lesion masks on fused PET/CT images. Inaccurate lesion regions were manually adjusted before feature extraction. All lesion voxels were included to evaluate whole-patient tumor burden. Tumor SUV_max_ and SUV_mean_ were calculated within the final lesion masks. Tumor volume was calculated as the number of lesion voxels multiplied by the volume of a single voxel. MTV was defined using a threshold of 41% of the lesion SUV_max_, and TLG was calculated as MTV multiplied by lesion SUV_mean_.

**Figure 2:**
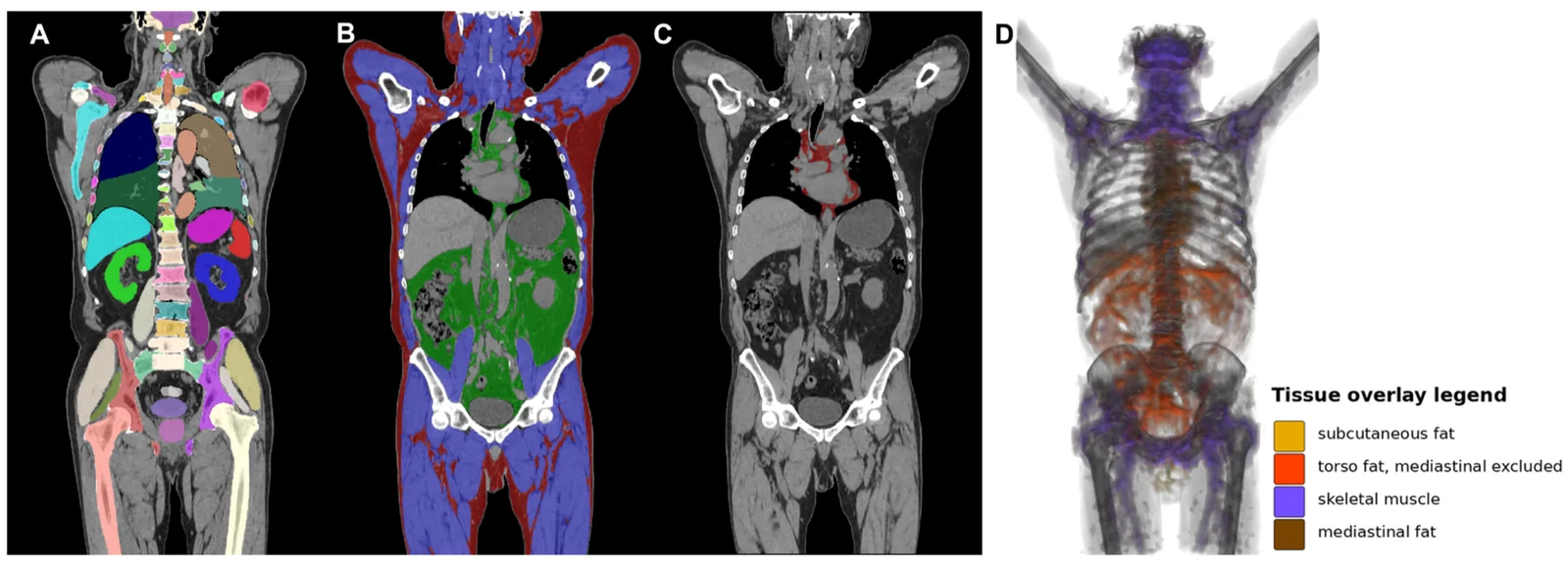
Representative PET/CT segmentation for organ and body composition analysis. (**A**) Multiorgan segmentation on coronal CT images. (**B**) CT-derived body composition segmentation, including SAT, SM, and TF. (**C**) Detailed visualization of MedF segmentation. (**D**) Three-dimensional rendering of the segmented body composition compartments. SAT, subcutaneous adipose tissue; SM, skeletal muscle; TF, torso fat; MedF, mediastinal fat.

Body composition was segmented automatically from CT images using the standard pretrained TotalSegmentator model [10]. The “tissue types” task was used to generate three-dimensional masks for subcutaneous adipose tissue (SAT), torso fat (TF), and skeletal muscle (SM). Segmentation was performed across all axial CT slices within the PET/CT acquisition range from the skull base to the mid-thigh. Thus, the reported SAT, TF and SM measurements represent three-dimensional tissue volumes within the acquired field of view rather than single-level surrogate measurements or estimates of total-body tissue mass. Mediastinal fat (MedF) was defined as TF voxels located between the fourth and eighth thoracic vertebrae. All masks were visually reviewed and manually corrected by two experienced nuclear medicine physicians. The schematic diagram of the segmentation results is shown in Figure 2.

Multiorgan segmentation was performed on CT images using TotalSegmentator. The segmented organs included the spleen, liver, bilateral kidneys, bilateral adrenal glands, stomach, pancreas, thyroid gland, heart, aorta, spinal cord and bilateral lungs. Organ masks from CT images were mapped to PET image space using nearest-neighbor interpolation. To reduce interference from tumor uptake, tumor lesion voxels were removed from the organ masks before organ SUV extraction. SUV_mean_ and volume were then calculated for each organ using the lesion-excluded organ masks.

Brain regions were segmented on PET images using another artificial intelligence-assisted nnU-Net-based model. Training labels were generated using atlas propagation. The MNI ICBM 152 template and anatomical parcellation were used as the reference atlas. The label set contained 51 anatomical regions, including bilateral cortical regions, deep gray matter nuclei, cerebellum, brainstem and ventricular structures. The resulting brain region masks were visually checked for anatomical matching by an experienced nuclear medicine physician. SUV_mean_ was then extracted for each brain region from the final masks.

For longitudinal analysis, the pretreatment PET/CT scan was used as the baseline measurement, and the follow-up scan was used as the post-treatment measurement. Delta values were defined as post-treatment values minus baseline values. A negative volume delta indicated shrinkage of the lesion or tissue after treatment, whereas a positive value indicated relative stability or enlargement.

### 2.5. Metabolic Network Construction

The network included 16 SUV_mean_-based nodes, consisting of the spleen, liver, stomach, pancreas, adrenal gland, thyroid gland, heart, aorta, spinal cord, lung, SAT, SM, TF, MedF, rostral anterior cingulate cortex (rACC), and medial orbitofrontal cortex (mOFC). Lesion-related metrics, including lesion SUV_max_, MTV, and TLG, were excluded because the network analysis focused on non-lesion systemic metabolism. Bilateral kidney SUV_mean_ was excluded to reduce the potential influence of urinary FDG excretion, and whole-brain SUV_mean_ was excluded because regional brain SUV_mean_ values were analyzed separately. Owing to missing brain data for one patient, 52 complete cases were available at baseline and 53 after treatment. The mOFC and rACC were selected as brain network nodes because they capture complementary aspects of food-related reward and regulatory processing. The mOFC is involved in encoding food reward value and subjective hedonic experience, whereas the rACC contributes to cognitive control, motivational regulation, and obesity-related changes in brain glucose metabolism. These regions therefore provided biologically relevant nodes for examining relationships among body composition, organ metabolism, and systemic metabolic remodeling [11,12,13].

A baseline reference partial-correlation network was constructed from 52 patients with complete baseline data. Partial correlations were obtained from the inverse of the node-wise correlation matrix after a ridge term of 1 × 10^−6^ was added to the diagonal for numerical stability. Individual edge perturbation was quantified using the Z-score of the correlation coefficient (ZCC), adapted from the sample-specific network framework of Liu et al. [14]: ZCC_ij = (Ptb_ij − Ref_ij)/[(1 − Ref_ij^2^)/(n_ref − 1)], where Ref_ij and Ptb_ij denote the reference and perturbed partial-correlation coefficients, respectively, and n_ref is the reference sample size. For baseline ZCC, the index patient was excluded from the baseline reference cohort (n_ref = 51), and that patient’s baseline observation was then added to construct the perturbed network, thereby preventing self-inclusion. For follow-up ZCC, the follow-up observation was added to the fixed complete baseline reference network (n_ref = 52). Both baseline and follow-up STR were therefore standardized perturbation measures anchored to the same baseline population. Edge strength was defined as |ZCC_ij|, node STR as the sum of |ZCC_ij| across all edges connected to a node, and total STR as the sum of all node STR values. Delta STR was calculated as follow-up STR minus baseline STR and represented the within-patient change in standardized network perturbation.

### 2.6. Statistical Analysis

Statistical analyses were performed using Python 3.13.12 and SPSS Statistics 27.0. Continuous variables were expressed as mean ± standard deviation, and categorical variables were presented as *n* (percentage). Two-sided *p* values < 0.05 were considered statistically significant. Within-patient comparisons between baseline and follow-up measurements were performed using two-sided Wilcoxon signed-rank tests. The Benjamini–Hochberg procedure was applied separately across the eight body-composition comparisons and the STR comparisons. Kaplan–Meier curves were generated for survival comparisons. Survival curves were compared using log-rank tests. Univariable and multivariable Cox proportional hazards models were used to estimate hazard ratios (HRs) and 95% confidence intervals (CIs). The proportional hazards assumption was assessed for Cox models. Continuous PET/CT-derived imaging variables were retained on their continuous scale in the Cox regression analyses. Median-based grouping was used only for descriptive Kaplan–Meier visualization. The Benjamini–Hochberg procedure was used to control the false discovery rate across the 22 imaging variables evaluated in the univariable Cox analyses. Potential nonlinearity in the association between delta TF volume and PFS was assessed using restricted cubic splines with knots at the 10th, 50th, and 90th percentiles.

## 3. Results

### 3.1. Patient Characteristics

The study included 53 patients with lung cancer who underwent both baseline and post-treatment PET/CT. The baseline clinical characteristics are summarized in Table 1. The cohort consisted of 40 male patients and 13 female patients, with a mean age of 65.06 ± 7.93 years and a mean BMI of 25.19 ± 3.03 kg/m^2^. Regarding tumor stage, T2 was the most frequent T stage (*n* = 19, 35.8%), followed by T3 (*n* = 15, 28.3%), T4 (*n* = 12, 22.6%), and T1 (*n* = 7, 13.2%). Most patients had nodal involvement, with N2 and N3 disease observed in 23 and 18 patients, respectively. Distant metastasis was present in 15 patients. The pathological subtypes included adenocarcinoma (ADC) in 22 patients, squamous cell carcinoma (SCC) in 13 patients and small-cell lung cancer (SCLC) in 18 patients. Treatment modalities included targeted therapy alone in 14 patients (26.4%), chemotherapy plus immunotherapy in 33 patients (62.3%), and targeted therapy plus immunotherapy in six patients (11.3%). The median interval from baseline to follow-up PET/CT was 110 days (IQR, 83.5–252 days; range, 58–829 days). The median interval from treatment initiation to follow-up PET/CT was 101 days (IQR, 78–255 days; range, 48–816 days). The median PFS was 7 months. During follow-up, 31 patients experienced disease progression or death, whereas 22 patients were censored without progression or death.

### 3.2. Changes in Body Composition Parameters After Treatment

**Figure 3:**
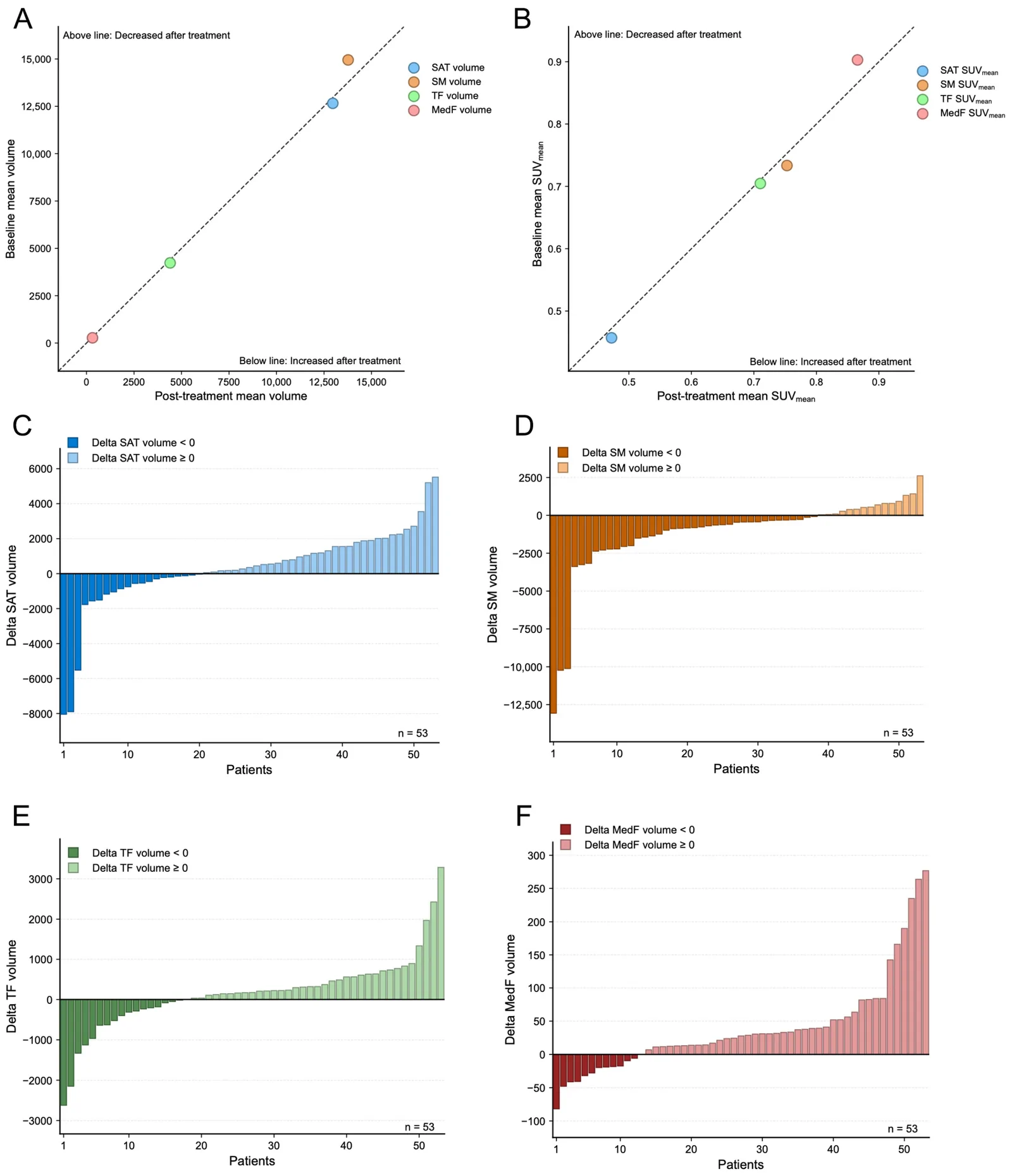
Longitudinal changes in PET/CT-derived body composition characteristics after treatment. The dashed line represents the line of identity (y = x), indicating equal pre- and post-treatment mean values. (**A**,**B**) Scatter plots comparing baseline and post-treatment mean values of body composition SUV_mean_ and volume. (**C**–**F**) Waterfall plots showing inter-patient heterogeneity in Delta SAT volume, Delta SM volume, Delta TF volume and Delta MedF volume. SAT, subcutaneous adipose tissue; SM, skeletal muscle; TF, torso fat; MedF, mediastinal fat.

Longitudinal changes in PET/CT-derived body composition parameters are summarized in Supplementary Table S1 and illustrated in Figure 3. None of the four SUV_mean_ parameters differed significantly between baseline and follow-up after FDR adjustment. Among the volume parameters, SM volume decreased from 14,949.02 ± 2928.38 at baseline to 13,768.29 ± 2911.71 at follow-up (*p* < 0.001; FDR-adjusted *p* = 0.001), whereas MedF volume increased from 275.38 ± 148.90 to 315.17 ± 182.69 (*p* < 0.001; FDR-adjusted *p* < 0.001). SAT volume increased from 12,658.80 ± 4489.09 to 12,962.76 ± 5114.84, but the difference did not remain significant after FDR adjustment (*p* = 0.031; FDR-adjusted *p* = 0.083). Similarly, TF volume increased from 4231.64 ± 1666.25 to 4400.54 ± 1648.38, without statistical significance after FDR adjustment (*p* = 0.043; FDR-adjusted *p* = 0.086). Thus, the most consistent longitudinal body composition changes were a decrease in SM volume and an increase in MedF volume.

Figure 3A and Figure 3B show cohort-level comparisons of baseline and post-treatment mean values for SUV_mean_ and volume parameters, respectively. Each point represents the intersection of the baseline mean value and post-treatment mean value for one body composition parameter. Points above the diagonal line indicate parameters that decreased after treatment, whereas points below the diagonal line indicate parameters that increased after treatment. Compared with SUV_mean_ parameters, volume parameters showed more evident post-treatment shifts. Waterfall plots demonstrated marked inter-patient heterogeneity in Delta SAT volume, Delta SM volume, Delta TF volume, and Delta MedF volume, indicating variable patterns of longitudinal body composition change across patients.

### 3.3. Univariable Cox Regression Analysis

Univariable Cox regression analysis was performed to evaluate the associations of clinical variables, PET/CT-derived body composition parameters, and tumor-related metabolic parameters with PFS. Clinical variables and PET/CT-derived variables associated with PFS are shown in Table 2, while the complete results are provided in Supplementary Table S2. In the continuous univariable Cox analyses, delta TF volume was not associated with PFS when modeled per 1-SD increase (HR, 0.81; 95% CI, 0.55–1.21; *p* = 0.304; FDR-adjusted *p* = 0.742). Baseline MedF volume and baseline lesion SUVmax had nominal *p* values below 0.05, but neither association remained significant after FDR adjustment. None of the 22 imaging variables met the FDR-adjusted significance threshold (Table S2).

Among the 53 patients, 29 underwent follow-up PET/CT before disease progression and were included in the post-PET/CT analysis. During subsequent follow-up, 12 patients experienced a PFS event, and 17 were censored. In the Cox regression analysis with PFS measured from the follow-up PET/CT date, delta TF volume was not significantly associated with subsequent PFS when analyzed per 1-SD increase (HR, 1.84; 95% CI, 0.91–3.68; *p* = 0.087; Table 3).

### 3.4. Kaplan–Meier Survival Analysis of Prognostic Variables

**Figure 4:**
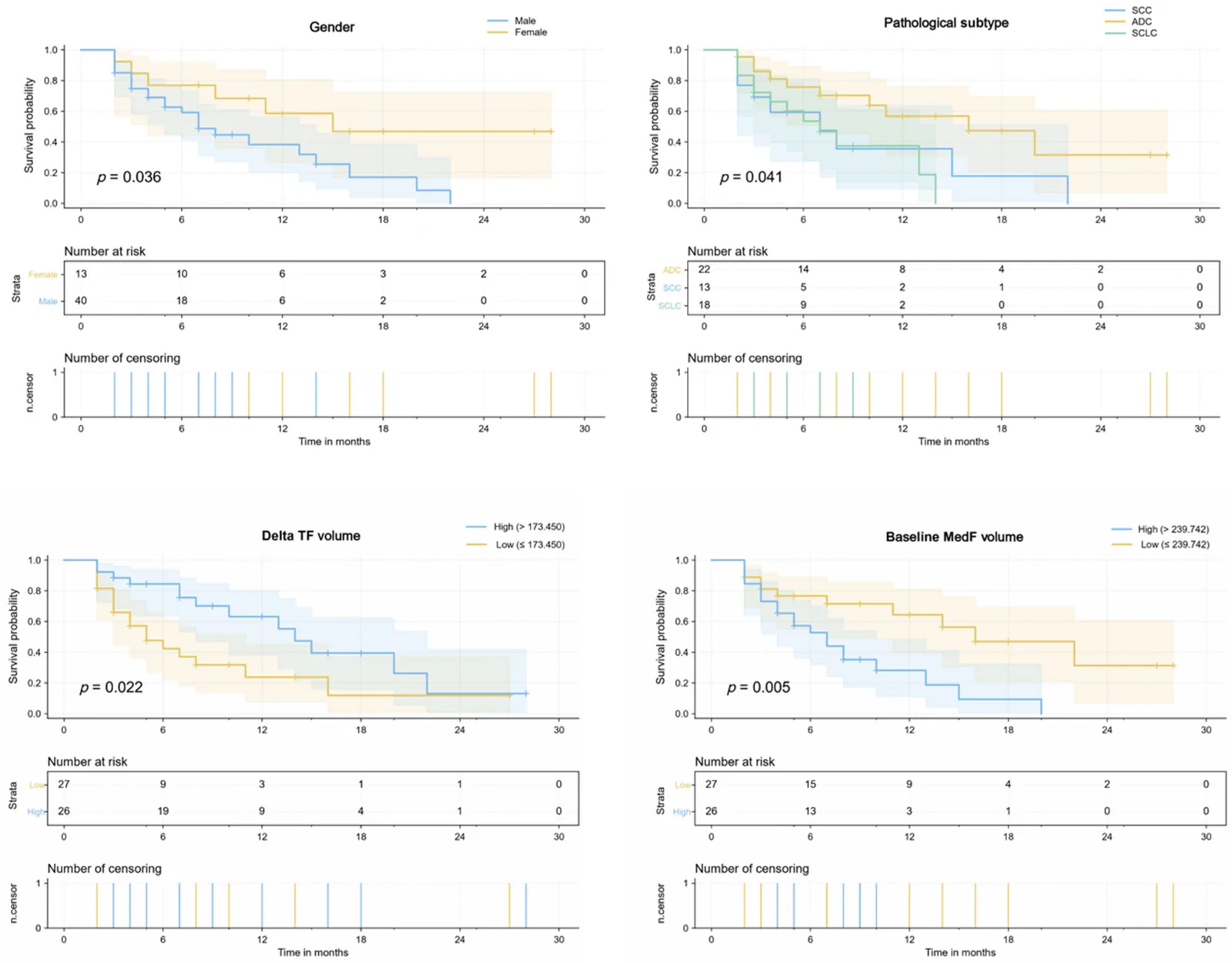
Kaplan–Meier survival analysis of prognostic variables. Kaplan–Meier curves are shown for sex, pathological subtype, delta TF volume, and baseline MedF volume. These variables were evaluated using the log-rank test. The shaded areas represent the 95% confidence intervals for the corresponding Kaplan–Meier curves. ADC, adenocarcinoma; SCC, squamous cell carcinoma; SCLC, small-cell lung cancer; TF, torso fat; MedF, mediastinal fat.

Kaplan–Meier survival analysis was performed for clinical and body composition-derived variables. As shown in Figure 4, sex, pathological subtype, delta TF volume, and baseline MedF volume showed significant differences in PFS by the log-rank test.

Female patients had longer PFS than male patients (*p* = 0.036). PFS also differed significantly among pathological subtypes (*p* = 0.041). Patients with SCC and SCLC showed poorer survival outcomes. For body composition parameters, patients with high delta TF volume had significantly longer PFS (*p* = 0.022). In contrast, patients with high baseline MedF volume had significantly shorter PFS (*p* = 0.005).

### 3.5. Multivariable Cox Regression Analysis

Given the study focus on longitudinal changes in adiposity, an exploratory multivariable Cox model was used to evaluate the adjusted association between delta TF volume and PFS. Three models were constructed, as shown in Table 4.

Model 1 included sex, pathological subtype, M stage, and baseline TLG, and none of these variables was significantly associated with PFS. In Model 2, baseline MedF volume was not significantly associated with PFS after adjustment for the variables included in Model 1 (HR, 1.14; 95% CI, 0.81–1.60; *p* = 0.452), and none of the other variables reached statistical significance. In Model 3, each 1-SD increase in delta TF volume was associated with a lower hazard of a PFS event (HR, 0.64; 95% CI, 0.43–0.97; *p* = 0.033). SCLC versus NSCLC was also associated with PFS in Model 3 (HR, 2.93; 95% CI, 1.23–6.98; *p* = 0.015).

Restricted cubic spline analyses showed no evidence of departure from linearity in the association between delta TF volume and PFS in either the full-cohort adjusted model (*p* = 0.952; Figure S1A) or the post-PET/CT landmark model (*p* = 0.695; Figure S1B). Delta TF volume was therefore retained as a linear continuous term in the Cox models.

### 3.6. Treatment-Related Remodeling of the Metabolic Network

**Figure 5:**
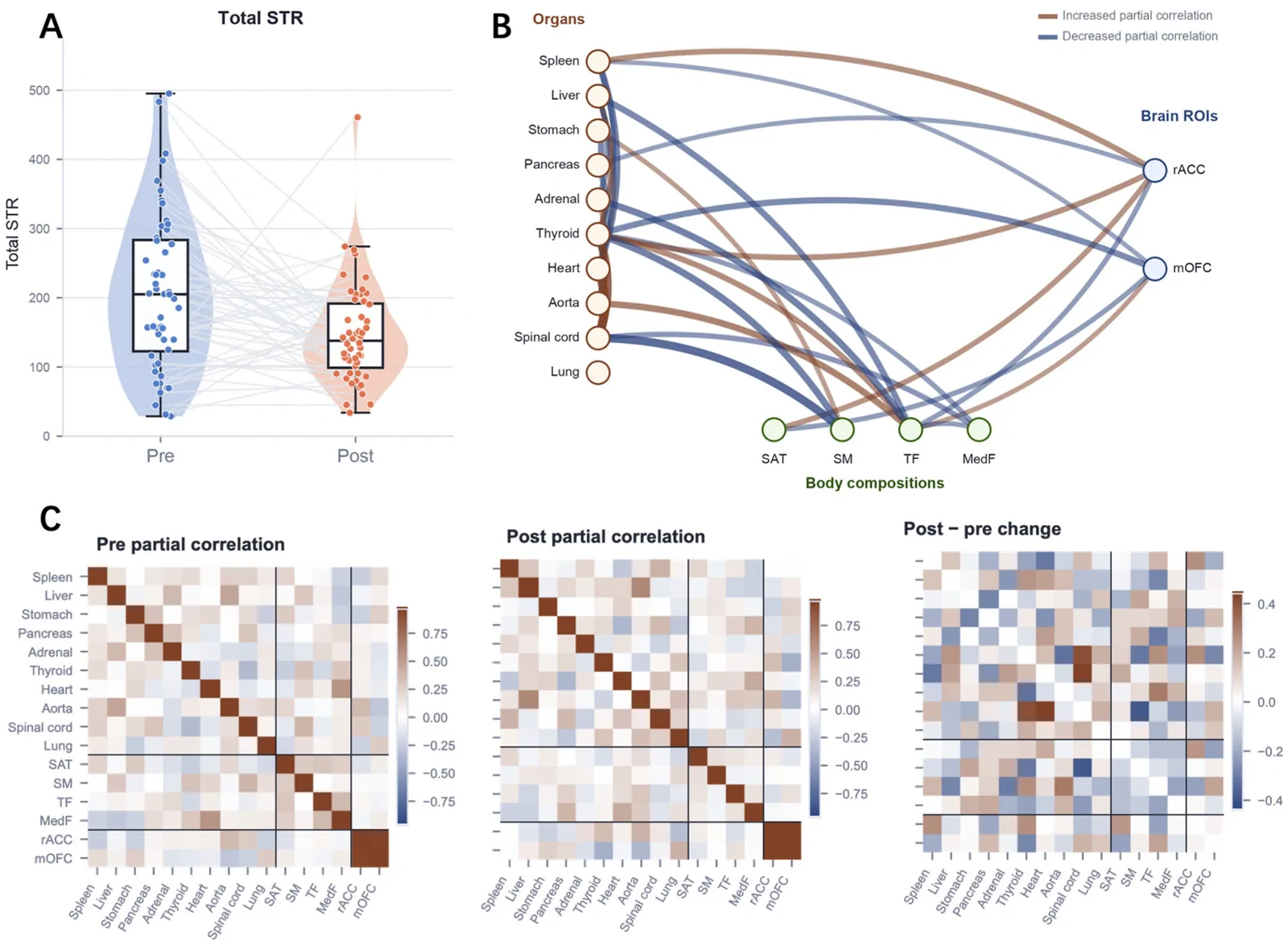
Treatment-related remodeling of the metabolic network. (**A**) Comparison of total STR between pre-treatment and post-treatment scans. (**B**) Network diagram showing treatment-related changes in inter-node partial correlations. (**C**) Group-level partial correlation heatmaps of the metabolic network. SAT, subcutaneous adipose tissue; SM, skeletal muscle; TF, torso fat; MedF, mediastinal fat; rACC, rostral anterior cingulate cortex; mOFC, medial orbitofrontal cortex; STR, strength of network perturbation.

To evaluate treatment-related systemic metabolic remodeling, total STR was calculated to quantify the overall perturbation of the brain-organ-body composition metabolic network. As shown in Figure 5A, under the predefined sample-specific network construction, total STR was lower at follow-up than at baseline (208.15 ± 113.40 vs. 147.19 ± 73.48, *p* = 0.002). Node-level analysis showed that the treatment-related decrease in STR was most evident in the mOFC, heart, aorta, rACC, MedF, and liver (Figure 5B, Supplementary Table S3). The mOFC showed the largest decrease in node STR, followed by several cardiovascular, metabolic, and body composition-related nodes. These findings suggest that the reduction in network perturbation after treatment involved both peripheral metabolic organs and metabolism-related brain regions. At the group level, partial correlation heatmaps showed treatment-related remodeling of the metabolic network (Figure 5C). The pre-treatment and post-treatment networks showed different patterns of inter-node metabolic associations. The post-pre change matrix further showed that these changes involved multiple edges across organs, body composition compartments, and brain regions.

The association between total STR and PFS was evaluated (Supplementary Figure S2 and Table S4). In the survival analyses, baseline total STR was not associated with PFS measured from baseline PET/CT (HR, 0.93; 95% CI, 0.65–1.35; *p* = 0.720). Follow-up total STR (HR, 1.01; 95% CI, 0.59–1.74; *p* = 0.961) and delta total STR (HR, 1.11; 95% CI, 0.66–1.86; *p* = 0.686) were not associated with subsequent PFS. None of the three associations met the FDR-adjusted significance threshold (Supplementary Table S4).

## 4. Discussion

This retrospective longitudinal PET/CT study investigated associations between metabolism-related imaging biomarkers and survival outcomes in patients with lung cancer receiving systemic therapy. Patients with an increase in TF volume after systemic therapy had significantly longer PFS. This association remained significant after adjustment for sex, pathological subtype, M stage, and baseline TLG. In addition, higher baseline MedF volume was associated with shorter PFS in univariable Cox and Kaplan–Meier analyses, but this association was markedly attenuated and was no longer significant after multivariable adjustment. Finally, exploratory network analysis showed a significant decrease in total STR after treatment, indicating reduced whole-body metabolic network perturbation in patients with lung cancer. However, neither post-treatment total STR nor change in total STR was significantly associated with PFS. Overall, longitudinal change in TF volume showed an association with PFS after adjustment for the selected clinical factors and baseline tumor metabolic burden.

The findings from our paired PET/CT analysis showed marked changes in body composition before and after treatment. After treatment, the volumes of SAT, TF and MedF increased, whereas SM volume decreased. This pattern suggests that fat accumulation and skeletal muscle loss may coexist, rather than reflecting a single direction of tissue depletion. Previous studies have shown that the core feature of cancer cachexia is ongoing SM loss, with or without fat loss [5]. Imaging studies have also suggested that patients with advanced cancer may show a trajectory of muscle loss with a transient increase in fat, resulting in a body composition phenotype characterized by increased adiposity and reduced skeletal muscle quantity [15].

SM loss may reflect persistent protein catabolism, inflammatory activation, reduced physical activity, and treatment-related muscle toxicity during therapy [15]. Previous studies in NSCLC have shown that early SM loss during chemotherapy is associated with poorer survival [16]. Lee et al. reported that patients with ADC had net SM loss during the disease course, and that those with rapid muscle loss had significantly shorter overall survival (OS) [17]. In contrast to SM loss, an increase in fat volume after treatment may reflect preserved energy reserve, attenuation of tumor-related catabolism, or improved nutritional status among patients who benefited from treatment. Tao et al. found that increased adipose tissue was associated with better OS in patients with NSCLC, independent of SM [18]. Patel et al. analyzed weight change during treatment in patients with advanced NSCLC and found that weight gain during therapy may be an early marker of clinical benefit [19]. These findings suggest that preservation of body weight or fat reserve during treatment may reflect not only nutritional status, but also tumor control, reduced systemic inflammation, and attenuation of catabolism. The clinical interpretation of adipose tissue as an imaging biomarker therefore requires caution. In addition to clinical studies suggesting that adipose tissue may be a favorable factor [18,20], some studies have reported longer survival in patients with higher BMI receiving immune checkpoint inhibitors, a phenomenon commonly referred to as the obesity paradox [2,21]. Experimental evidence has also shown that obesity may promote chronic inflammation and immune dysfunction, and may modify responses to PD-1/PD-L1 blockade under certain conditions [3]. The association between increased TF volume and longer PFS may reflect several related clinical processes, including treatment response, recovery from cancer-related catabolism, and improvement in general condition. Delta TF volume may therefore serve as an imaging-derived marker of the patient’s clinical trajectory rather than an isolated determinant of prognosis. It should be noted that the observed decrease in skeletal muscle volume represents a reduction in skeletal muscle quantity but does not directly indicate impaired muscle quality or strength. Because muscle strength and physical performance were not assessed, these imaging findings should not be interpreted as a diagnosis of sarcopenia according to the European Working Group on Sarcopenia in Older People 2 (EWGSOP2) framework [6].

Baseline MedF volume showed a different prognostic pattern. Higher baseline MedF volume was associated with shorter PFS in univariable Cox and Kaplan–Meier analyses. However, this association was no longer statistically significant after adjustment for clinical characteristics and baseline TLG. The apparent association may therefore be partly explained by differences in baseline clinical features or tumor-related metabolic status. Although the study population differed from ours, a previous cohort of patients with resectable lung cancer similarly found no significant association between mediastinal adipose tissue and long-term survival [22]. MedF volume may reflect local thoracic fat accumulation and may also be related to the metabolic and inflammatory environment around the mediastinum. Nevertheless, our findings do not support baseline MedF volume as an independent prognostic biomarker. Future studies incorporating inflammatory markers, lipidomic features, PD-L1 expression, driver gene mutations, and detailed treatment information may help clarify its biological relevance.

Finally, the exploratory metabolic network analysis offered an additional perspective on systemic metabolic remodeling during therapy. Total STR decreased significantly after treatment, with prominent node-level changes involving the mOFC, rACC, cardiovascular nodes, MedF, and liver. Concurrent alterations across central brain regions, peripheral metabolic organs, and body composition compartments suggest that systemic therapy may be accompanied by reorganization of coordinated metabolism across the body. Nevertheless, total STR was not significantly associated with PFS in this cohort. Therefore, total STR was not validated as a significant prognostic marker in this cohort. However, the direction of these associations was consistent with the biological interpretation that lower residual metabolic network perturbation after treatment may reflect a more stable systemic metabolic state and may be related to more favorable disease control.

This study has several limitations. First, requiring both baseline and follow-up PET/CT may have introduced potential selection bias. Therefore, the study cohort primarily represents patients with primary lung cancer who received the specified treatments and completed serial PET/CT examinations. Second, this was a single-center retrospective study with a relatively small cohort. Although paired PET/CT enabled longitudinal assessment of body composition within the same patients, the sample size may have limited the precision of effect estimates and the stability of multivariable models. Third, the cohort included patients with different pathological subtypes and systemic treatment regimens. ECOG performance status, smoking history, treatment line, PD-L1 expression, detailed driver-mutation profiles, and formal nutritional assessments were not systematically available and could not be incorporated into the multivariable models. Residual confounding from these unmeasured clinical factors should therefore be considered when interpreting the adjusted associations. Accordingly, the findings characterize longitudinal imaging phenotypes across a mixed systemic-therapy cohort rather than treatment-specific effects. Fourth, the cohort included 13 female and 40 male patients. This imbalance limited the precision of sex-related estimates and the ability to perform robust sex-stratified analyses. Finally, the relatively recent establishment of the cohort and limited follow-up duration precluded a reliable overall survival (OS) analysis. Longer follow-up is required to evaluate the associations of the imaging biomarkers with OS.

## 5. Conclusions

Paired PET/CT identified longitudinal changes in body composition and systemic metabolic network measures during systemic therapy. Delta TF volume was associated with PFS in an exploratory full-cohort adjusted model, although this association was not statistically significant in the post-PET/CT analysis. Larger prospective studies with predefined imaging time points are needed to determine its prognostic relevance.

## Figures and Tables

**Table 1:** Baseline clinical characteristics of the study cohort.

Characteristics	Group	*n* = 53
Sex	Female	13 (24.5%)
	Male	40 (75.5%)
Age	Total	65.06 ± 7.93
	Female	64.00 ± 2.63
	Male	65.40 ± 1.18
BMI	Total	25.19 ± 3.03
	Female	23.62 ± 0.63
	Male	25.70 ± 0.49
T stage	T1	7 (13.2%)
	T2	19 (35.8%)
	T3	15 (28.3%)
	T4	12 (22.6%)
N stage	N0	6 (11.3%)
	N1	6 (11.3%)
	N2	23 (43.4%)
	N3	18 (34.0%)
M stage	M0	38 (71.7%)
	M1	15 (28.3%)
Pathological subtype	ADC	22 (41.5%)
	SCC	13 (24.5%)
	SCLC	18 (34.0%)
Median PFS, months	7	
Progression or death during follow-up	1	31 (58.5%)
	0	22 (41.5%)
Treatment modality	Targeted therapy	14 (26.4%)
	Chemotherapy + immunotherapy	33 (62.3%)
	Targeted therapy + immunotherapy	6 (11.3%)
Baseline-to-follow-up PET/CT interval, days	Median (IQR), 110 (83.50, 264.00)	Range, 58–829
Treatment-to-follow-up PET/CT interval, days	Median (IQR), 101 (78.00–255.00)	Range, 48–816

Note: Data are presented as mean ± standard deviation, median (interquartile range), or number (percentage). BMI, body mass index; PFS, progression-free survival. Disease progression or death was coded as an event (event = 1), whereas patients without progression or death by the follow-up cutoff date were censored (event = 0).

**Table 2:** Univariable Cox regression analysis of factors associated with PFS.

Variable	Comparison	HR (95% CI)	*p* Value	FDR *p* Value
Sex	Male vs. Female	2.56 (1.03–6.39)	0.044	
Pathological subtype	SCC vs. ADC	2.43 (0.98–6.03)	0.056	
	SCLC vs. ADC	2.80 (1.15–6.84)	0.024	
	SCC vs. SCLC	0.87 (0.35–2.12)	0.754	
M stage	M1 vs. M0	1.42 (0.68–2.95)	0.349	
Baseline lesion SUVmax	Per 1-SD increase	1.47 (1.04–2.06)	0.028	0.429
Baseline TLG	Per 1-SD increase	1.24 (0.86–1.78)	0.243	0.742
Baseline MedF volume	Per 1-SD increase	1.39 (1.02–1.90)	0.039	0.429
Delta TF volume	Per 1-SD increase	0.81 (0.55–1.21)	0.304	0.742

Note: PFS, progression-free survival; HR, hazard ratio; CI, confidence interval; SCLC, small-cell lung cancer; TF, torso fat.

**Table 3:** Post-PET/CT landmark-type Cox regression analysis of delta TF volume.

Characteristics	Patients	PFS Events	HR	95% CI	*p* Value
Delta TF volume	29	12	1.84	0.91–3.68	0.087

Note: PFS was measured from the follow-up PET/CT date to the recorded PFS event or censoring. CI = confidence interval.

**Table 4:** Exploratory multivariable Cox regression models for PFS.

Model	Variable	Comparison	HR (95% CI)	*p* Value
Model 1	Sex	Male vs. Female	2.03 (0.79–5.23)	0.141
	Pathological subtype	SCLC vs. NSCLC	2.22 (0.98–5.03)	0.056
	M stage	M1 vs. M0	1.27 (0.59–2.71)	0.545
	Baseline TLG	Per 1-SD increase	1.28 (0.84–1.93)	0.247
Model 2	Gender	Male vs. Female	1.84 (0.69–4.93)	0.227
	Pathological subtype	SCLC vs. NSCLC	2.08 (0.90–4.82)	0.086
	M stage	M1 vs. M0	1.23 (0.57–2.65)	0.605
	Baseline TLG	Per 1-SD increase	1.25 (0.82–1.90)	0.292
	Baseline MedF volume	Per 1-SD increase	1.14 (0.81–1.60)	0.452
Model 3	Gender	Male vs. Female	2.33 (0.89–6.08)	0.085
	Pathological subtype	SCLC vs. NSCLC	2.93 (1.23–6.98)	0.015
	M stage	M1 vs. M0	1.50 (0.67–3.35)	0.324
	Baseline TLG	Per 1-SD increase	1.34 (0.90–2.01)	0.153
	Delta TF volume	Per 1-SD increase	0.64 (0.43–0.97)	0.033

Note. NSCLC, non-small-cell lung cancer; SCLC, small-cell lung cancer; CI, confidence interval; HR, hazard ratio; TF, torso fat; TLG, total lesion glycolysis.

## Data Availability

The datasets used during the current study are available from the corresponding author on reasonable request.

## Supplementary Materials

The following supporting information can be downloaded at: https://www.mdpi.com/article/10.3390/diagnostics16182930/s1, Figure S1: Restricted cubic spline analyses of the association between delta TF volume and PFS. (A) Full-cohort analysis adjusted for sex, pathological subtype, M stage, and baseline total lesion glycolysis. (B) Post-PET/CT analysis with PFS measured from the follow-up PET/CT date; Figure S2: Forest plot showing the associations of total STR measures with PFS. Baseline total STR was evaluated using PFS measured from baseline PET/CT, whereas follow-up and delta total STR were evaluated using PFS measured from follow-up PET/CT. Points represent hazard ratios per 1-SD increase, and horizontal lines represent 95% confidence intervals; Table S2: Univariable Cox regression analysis of factors associated with PFS; Table S3: Node-level changes in STR after treatment; Table S4: Associations between total STR measures and PFS.
