# Supplementary material for: Longitudinal PET/CT-Derived Body Composition Changes During Systemic Therapy in Patients with Lung Cancer
Source: Diagnostics (Basel). 2026 Sep 10;16(18):2930. doi: 10.3390/diagnostics16182930 (PMC13605957; doi:10.3390/diagnostics16182930)
Supplement: Supplementary file 1 [file diagnostics-16-02930-s001.zip › diagnostics-4511799-supplementary.pdf]

Supplementary materials

Table S1. Longitudinal changes in PET/CT-derived body composition characteristics

Characteristics	Baseline value	Follow-up value	Delta value	*p* value	*FDR p value*
SAT SUV_{mean}	0.46 ± 0.09	0.47 ± 0.09	0.02 ± 0.08	0.444	0.507
SAT volume	12658.80 ± 4489.09	12962.76 ± 5114.84	303.96 ± 2378.13	0.031	0.083
SM SUV_{mean}	0.73 ± 0.12	0.753 ± 0.100	0.02 ± 0.12	0.258	0.345
SM volume	14949.02 ± 2928.38	13768.29 ± 2911.71	−1180.73 ± 2754.34	<0.001	0.001
TF SUV_{mean}	0.70 ± 0.13	0.71 ± 0.13	0.01 ± 0.12	0.663	0.849
TF volume	4231.64 ± 1666.25	4400.54 ± 1648.38	168.89 ± 921.42	0.043	0.086
MedF SUV_{mean}	0.90 ± 0.21	0.87 ± 0.17	-0.04 ± 0.19	0.177	0.233
MedF volume	275.38 ± 148.90	315.17 ± 182.69	39.78 ± 72.84	<0.001	<0.001

Note: Data are presented as mean ± SD. P values were obtained using two-sided Wilcoxon signed-rank tests. FDR-adjusted P values were calculated using the Benjamini–Hochberg procedure. SAT, subcutaneous adipose tissue; SM, skeletal muscle; TF, torso fat; MedF, mediastinal fat.

Table S2. Univariable Cox regression analysis of factors associated with PFS

Variable	Comparison	HR (95% CI)	*p* value	FDR-adjusted *p* value
Clinical characteristics				
Sex	Male vs. Female	2.56 (1.03–6.39)	0.044	
Age	Per 1-year increase	1.04 (0.99–1.09)	0.153	
BMI	Per 1-kg/m² increase	1.05 (0.92–1.20)	0.428	
Pathological subtype	SCC vs. ADC	2.43 (0.98–6.03)	0.056	
	SCLC vs. ADC	2.80 (1.15–6.84)	0.024	
	SCC vs. SCLC	0.87 (0.35–2.12)	0.754	
T stage	T2 vs. T1	0.85 (0.23–3.23)	0.815	
	T3 vs. T1	1.47 (0.41–5.32)	0.555	
	T4 vs. T1	1.43 (0.38–5.31)	0.594	
N stage	N1 vs. N0	1.04 (0.26–4.20)	0.952	
	N2 vs. N0	0.63 (0.20–1.95)	0.419	
	N3 vs. N0	0.80 (0.25–2.54)	0.704	
M stage	M1 vs. M0	1.42 (0.68–2.95)	0.349	
Body composition				
Baseline SAT SUVmean	Per 1-SD increase	1.21 (0.86–1.72)	0.275	0.742
Delta SAT SUVmean	Per 1-SD increase	1.06 (0.73–1.53)	0.769	0.961
Baseline SAT volume	Per 1-SD increase	0.98 (0.65–1.47)	0.912	0.961
Delta SAT volume	Per 1-SD increase	0.82 (0.56–1.19)	0.290	0.742
Baseline SM SUVmean	Per 1-SD increase	1.14 (0.80–1.63)	0.458	0.861
Delta SM SUVmean	Per 1-SD increase	0.96 (0.67–1.36)	0.811	0.961
Baseline SM volume	Per 1-SD increase	1.33 (0.95–1.88)	0.098	0.718
Delta SM volume	Per 1-SD increase	0.90 (0.59–1.37)	0.611	0.961
Baseline TF SUVmean	Per 1-SD increase	1.08 (0.76–1.54)	0.660	0.961
Delta TF SUVmean	Per 1-SD increase	0.99 (0.68–1.43)	0.937	0.961
Baseline TF volume	Per 1-SD increase	1.30 (0.90–1.90)	0.164	0.742
Delta TF volume	Per 1-SD increase	0.81 (0.55–1.21)	0.304	0.742
Baseline MedF SUVmean	Per 1-SD increase	0.90 (0.65–1.24)	0.509	0.861
Delta MedF SUVmean	Per 1-SD increase	0.99 (0.71–1.39)	0.961	0.961
Baseline MedF volume	Per 1-SD increase	1.39 (1.02–1.90)	0.039	0.429
Delta MedF volume	Per 1-SD increase	1.06 (0.74–1.53)	0.735	0.961
Lesion burden parameters				
Baseline lesion SUVmax	Per 1-SD increase	1.47 (1.04–2.06)	0.028	0.429
Delta lesion SUVmax	Per 1-SD increase	0.96 (0.67–1.38)	0.845	0.961
Baseline MTV	Per 1-SD increase	1.26 (0.87–1.82)	0.222	0.742
Delta MTV	Per 1-SD increase	0.87 (0.59–1.30)	0.502	0.861
Baseline TLG	Per 1-SD increase	1.24 (0.86–1.78)	0.243	0.742
Delta TLG	Per 1-SD increase	0.87 (0.59–1.30)	0.508	0.861

Note: ADC, adenocarcinoma; BMI, body mass index; CI, confidence interval; HR, hazard ratio; MedF, mediastinal fat; MTV, metabolic tumor volume; PFS, progression-free survival; SAT, subcutaneous adipose tissue; SCC, squamous cell carcinoma; SCLC, small-cell lung cancer; SM, skeletal muscle; SUV, standardized uptake value; TF, torso fat; TLG, total lesion glycolysis.

Table S3. Node-level changes in STR after treatment

Node	Pre STR mean	Post STR mean	Mean delta	*P* value	FDR-adjusted *p* value
SAT	13.136	10.228	-2.908	0.353	0.403
SM	13.096	11.807	-1.289	0.418	0.445
TF	13.863	10.148	-3.715	0.103	0.171
MedF	13.246	8.408	-4.839	0.006	0.023
Spleen	13.168	8.841	-4.328	0.124	0.171
Liver	13.636	8.252	-5.384	0.009	0.025
Stomach	12.658	10.449	-2.210	0.339	0.403
Pancreas	13.500	10.140	-3.360	0.119	0.171
Adrenal	11.860	10.117	-1.742	0.649	0.649
Thyroid	11.600	8.815	-2.785	0.103	0.171
Heart	13.838	8.183	-5.655	0.004	0.020
Aorta	13.107	7.896	-5.211	0.003	0.020
Lung	12.604	8.960	-3.644	0.022	0.051
Spinal cord	13.524	11.085	-2.440	0.128	0.171
rACC	12.821	7.268	-5.553	0.007	0.023
mOFC	12.492	6.597	-5.895	<0.001	0.007

Note: SAT, subcutaneous adipose tissue; SM, skeletal muscle; TF, torso fat; MedF, mediastinal fat; rACC, rostral anterior cingulate cortex; mOFC, medial orbitofrontal cortex.

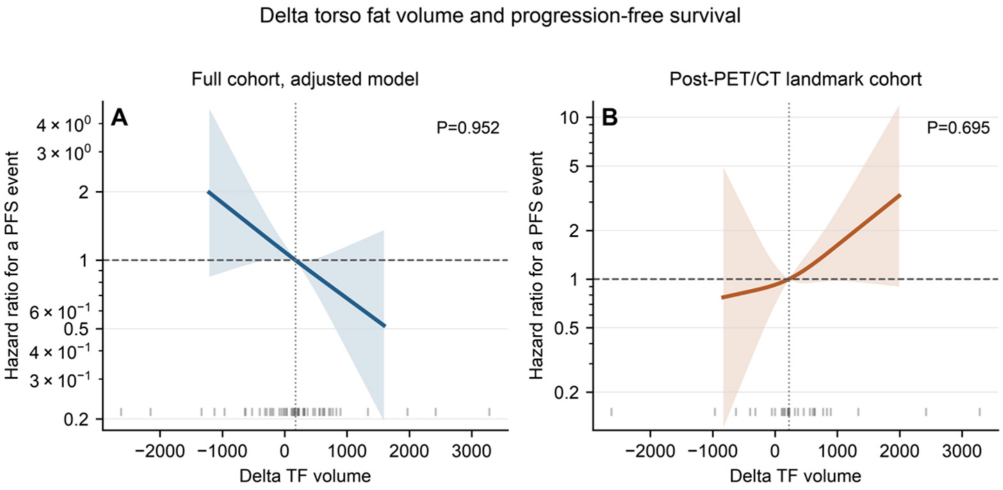

Figure S1. Restricted cubic spline analyses of the association between delta TF volume and PFS. (A) Full-cohort analysis adjusted for sex, pathological subtype, M stage, and baseline total lesion glycolysis. (B) Post-PET/CT analysis with PFS measured from the follow-up PET/CT date.

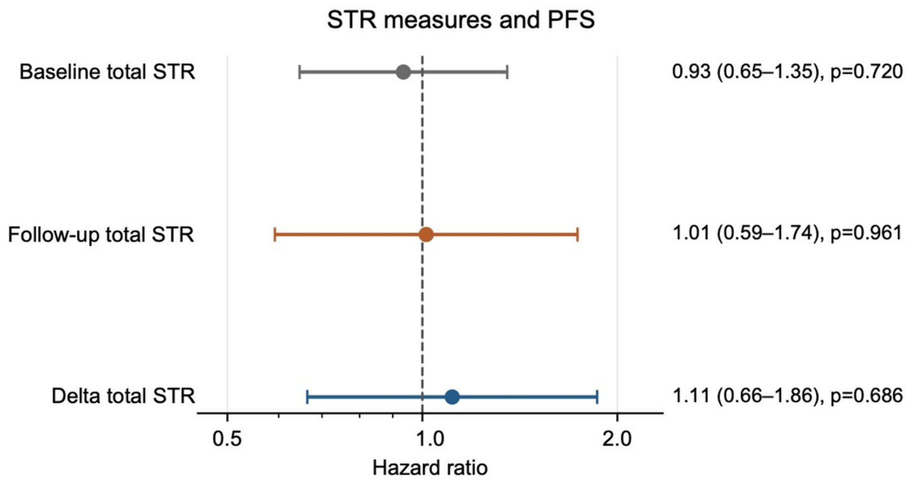

Figure S2. Forest plot showing the associations of total STR measures with PFS. Baseline total STR was evaluated using PFS measured from baseline PET/CT, whereas follow-up and delta total STR were evaluated using PFS measured from follow-up PET/CT. Points represent hazard ratios per 1-SD increase, and horizontal lines represent 95% confidence intervals.

Table S4. Associations between total STR measures and PFS

STR measure	PFS time origin	Comparison	HR (95% CI)	*p* value	FDR-adjusted *p* value
Baseline total STR	Baseline PET/CT	Per 1-SD increase	0.93 (0.65–1.35)	0.72	0.961
Follow-up total STR	Follow-up PET/CT	Per 1-SD increase	1.01 (0.59–1.74)	0.961	0.961
Delta total STR	Follow-up PET/CT	Per 1-SD increase	1.11 (0.66–1.86)	0.686	0.961

Note. HRs are reported per 1-SD increase. CI, confidence interval; FDR, false discovery rate; HR, hazard ratio; PFS, progression-free survival; STR, strength of network perturbation.
